# Design and methods of the mobile assessment of cognition, environment, and sleep (MACES) feasibility study in newly diagnosed breast cancer patients

**DOI:** 10.1038/s41598-024-58724-1

**Published:** 2024-04-09

**Authors:** Rebecca Derbes, Jonathan Hakun, Daniel Elbich, Lindsay Master, Sheri Berenbaum, Xuemei Huang, Orfeu M. Buxton, Anne-Marie Chang, Cristina I. Truica, Kathleen M. Sturgeon

**Affiliations:** 1 Department of Public Health Sciences, College of Medicine, Pennsylvania State University, Hershey, PA USA; 2 Penn State Milton S. Hershey Medical Center, Department of Neurology, H5508, College of Medicine, Pennsylvania State University, 500 University Drive, Hershey, PA H03717033 USA; 3 Department of Psychology, College of Liberal Arts, Pennsylvania State University, University Park, PA USA; 4 Center for Healthy Aging, The Pennsylvania State University, University Park, PA 16802 USA; 5 College of Medicine, Translational Brain Research Center, Pennsylvania State University, Hershey, PA 17033 USA; 6 Department of Biobehavioral Health, College of Health and Human Development, Pennsylvania State University, University Park, PA USA; 7 Division of Hematology and Oncology, Department of Medicine, Pennsylvania State University, Hershey, PA USA

**Keywords:** Neoplasm, Cognition, Sleep, Physical activity, Ecological momentary assessment, Endocrine therapy, Oncology, Signs and symptoms

## Abstract

Endocrine therapy (ET) for breast cancer treatment is associated with cognitive complaints, but their etiology is poorly understood. To address this, we developed and implemented an ambulatory assessment protocol consisting of wearable activity monitors, brief surveys of affect, context, and perceived impairments, and ultra-brief performance-based measures of cognition. Newly diagnosed, ER/PR+, stage 0-III, female breast cancer patients, were recruited. Ambulatory assessments were conducted on smart phones and wearable activity monitors were used to monitor sleep and physical activity. Participants were asked to complete five 7-day measurement bursts (one before starting ET and one each month for 4 consecutive months while on ET). We observed a consent rate of 36%, 27 women completed the study. Of the women that withdrew, 91% dropped prior to the midpoint of follow up. There were no significant differences in demographics, clinical breast cancer characteristics, sleep or physical activity patterns, or measures of cognition between women who completed versus withdrew. Women who did not complete the study provided fewer valid days of baseline data. In conclusion, while some women may be overwhelmed with their cancer diagnosis, we did not identify any predictive characteristics of women whom did not complete the study. This novel method enables the prospective study of psychological changes associated with cancer treatment, capturing a wide array of information about behavior, experience, and cognition, thus providing a picture of the lived experiences of cancer patients before and during exposure to ET.

## Introduction

Advances in cancer detection and treatment have increased the average five-year survival rate for breast cancer patients to 90%^1^. Endocrine therapy (ET) such as tamoxifen and/or aromatase inhibitors reduces the risk of breast cancer recurrence in patients with estrogen receptor positive tumors (60% of breast cancer)^2^, but is often accompanied by complaints of cognitive impairment (independent of chemotherapy)^3–5^. Endocrine therapy-induced adverse effects on cognition are common, clinically underreported, and challenging to manage. For a comprehensive review on the nature, incidence, risk factors, and underlying mechanisms of endocrine therapy-induced cognitive dysfunction, we refer readers to Haggstrom *et al*^5^*.* Cognitive impairment can adversely affect treatment adherence, activities of daily living, and quality of life for survivors^6^. The burden of cancer related cognitive impairment can manifest with a wide range of severity, and evidence suggests that cognitive impairment is an important factor relative to work ability, return to work, and work performance^7^.

The nature and etiology of cognitive impairment in patients receiving adjuvant therapies remains poorly understood. Endocrine therapy attenuates the availability of estrogen and interrupts estrogen signaling, which may alter regulatory systems in the brain^8,9^. Critically, studies linking patient-reported cognitive outcomes with performance-based measures have produced inconsistent results^10,11^, raising the possibility that patient complaints may reflect changes in psychosocial factors (e.g., occupational and social disconnection, stress) rather than the impact of ET on the brain^12,13^. Alternatively, given other studies showing estrogen effects on cognition related to the menstrual cycle, menopause, and hormone replacement therapy^8^, cognitive impairments associated with ET may not be adequately assessed because of insensitivity of standard, lab- or clinic-based neuropsychological tests to focal cognitive deficits that might emerge during treatment, and lack of baseline assessment and longitudinal follow-up^14,15^.

To remedy this issue, we developed an ambulatory assessment protocol that enables repeated assessment of multiple domains likely to be affected by ET, and of links across domains (e.g., mood or sleep effects on cognition). Assessments include brief surveys of affect/mood, context, and self-reported cognitive impairments, ultra-brief performance-based cognitive assessments, and passive sensing via wearable activity monitors. This ambulatory approach allows for high-resolution surveillance of cognition, context, and behavior over the course of patients’ lives where episodes of cognitive impairment naturally occur. The goal of our approach is to understand the time course of ET-related cognitive impairments and identify the contexts that are correlated with their onset, whether they be psychosocial (e.g., following exposure to an everyday stressor), behavioral/regulatory (e.g., a poor night’s sleep, high levels of sedentary behavior), or cognitive (e.g., subjective impairment reported during moments or days when objective deficits in cognitive performance are observed).

Ambulatory assessment of cognition has been used successfully in studies of aging^16^, chronic pain^17^, and breast cancer survivors^18,19^. Results of these studies suggest that ambulatory assessments may be more sensitive than conventional methods to cancer- and cancer treatment-associated cognitive impairment. Critically, ambulatory protocols can be intensive (e.g., involving 4 + assessments per day) and potentially burdensome to patients (e.g., requiring up to 20 min per day of testing), raising concerns about conducting such a study among newly diagnosed patients as they begin cancer treatment. Therefore, we report on (1) the design and (2) methodology of our study, as well as (3) consent, completion, and compliance rate of newly diagnosed patients with breast cancer. By comparing the characteristics of patients who completed our study with those who withdrew, we sought to provide guidance for future studies that hope to leverage similar design and methodology.

## Methods

### Study design overview

**Figure 1:**
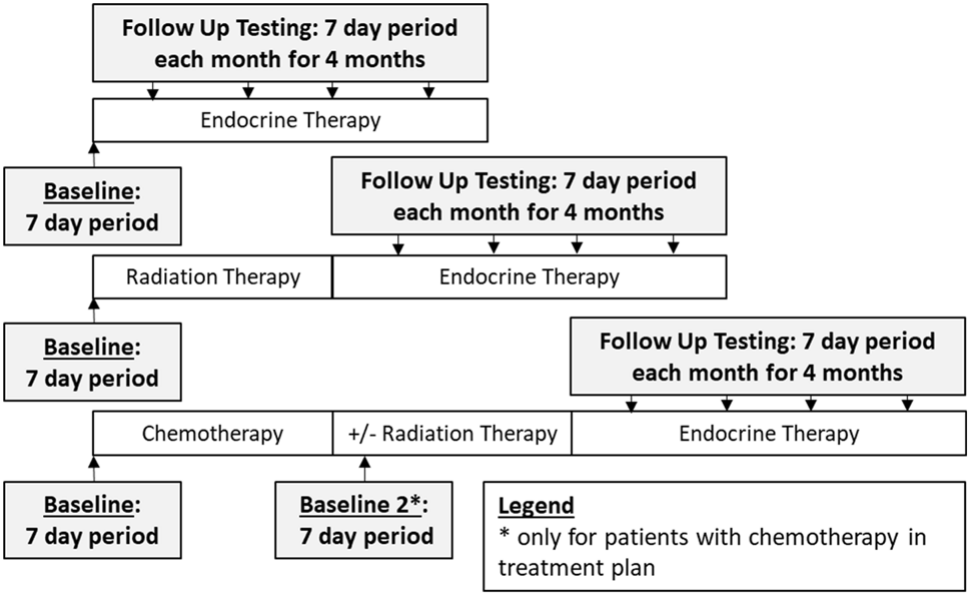
Study schema detailing when the 7-day data collection time points occurred for participants receiving endocrine therapy, radiation + endocrine therapy or chemotherapy ± radiation therapy and endocrine therapy.

The study was designed as a prospective observational study (Fig. 1). Each wave of data collection was conducted in a measurement burst design^20^. Measurement bursts involved self-report ecological momentary assessment (EMA) surveys, ambulatory cognitive assessments on smart phones, and continuous data on physical activity and sleep obtained from wrist- and hip-worn devices for a period of 7 days; details are provided below. EMA and ambulatory cognitive assessments were delivered on study-provided smart phones via a prototype of the Mobile Monitoring of Cognitive Change (M2C2) mobile platform. Participants were asked to complete 6 administrations per day of the EMA surveys and ambulatory cognitive assessments: 2 self-initiated administrations (one at waking “Wake-up Survey” and one before bedtime “Bedtime Survey”) and 4 notified “Beeped Surveys” that were pseudo-randomly triggered by the M2C2 platform. A high-frequency measurement burst design was chosen for the current study in order to assess the potential contextual factors that might contribute to the experience of cognitive impairment over the course of patients’ everyday lives. Such experience-sampling methods have been used with success in other populations, including breast cancer survivors^18,19^, to increase the sensitivity to temporally distributed events such as the frequency of perceived impairments. As with all experience-sampling methods, the choice of assessment frequency (e.g. number of assessments/day) entails striking a balance between sampling frequently enough to capture the phenomena of interest (e.g. a fleeting episode of forgetting, exposure to a stressor) and participant burden. We chose to sample with higher frequency (6 assessments/day) in order to generate a detailed picture of patients’ everyday lives in the sample. In addition, the duration of the burst protocol should consider sampling across a timeframe that represents an individual’s experience throughout their daily life, and thus, we chose to sample for 7 days to ensure we captured the various exposures and experiences that occur on both weekdays and weekends. A major aim of the study was to assess whether this frequency was tolerable and feasible and to leverage this information for the design of future studies in response to patient feedback. Wearable activity monitors were programmed to continuously collect data for the entire measurement burst.

Following documentation of written informed consent, the research coordinator provided the participant with the study devices (smartphone and activity monitors) and instructions. The participant was asked to complete baseline measurements and a demographic survey and then to return the devices either in person or via shipment using provided pre-paid label and packaging. During follow up, devices were provided and returned either in person or via shipment depending on coordination with the participant. Participants received retention materials over the course of the study (thank you cards following device return) and $60 upon completion of all study measurements. The study was approved by the Penn State College of Medicine and the Mount Nittany Medical Center Institutional Review Boards and we certify that the study was performed in accordance with the ethical standards as laid down in the 1964 Declaration of Helsinki and its later amendments or comparable ethical standards.

No data was available to determine sample size calculations for this population or our longitudinal outcomes. We targeted enrollment of 50 women in order to determine: rate of participation (percent of eligible patients who participated), and of completion of different phases of the protocol; amount and nature of attrition (within and across phases) and missing data; and contributors to retention, including demographics (e.g., age, race) and baseline characteristics (e.g., overall cognitive ability, chronic stress). The overall study goals and primary outcomes (cognitive changes with hormonal treatment) capitalize on our repeated assessments and acquisition of hypothesized moderators (e.g., sleep quality, physical activity, stress), to determine effect sizes for autoregressive multilevel mixed models; and structural equation modeling.

### Recruitment, screening and eligibility

The target population was women with newly diagnosed breast cancer, recruited at the Penn State Cancer Institute and the Mount Nittany Health, Cancer Care Partnership. Eligibility criteria are presented in Table 1. Study staff confirmed eligibility criteria through electronic medical review and patient-self report. Staff approached screened patients at either surgical post-op following tumor resection or initial medical oncology or radiology consults. Observed reasons for ineligibility are presented in Table 2.

**Table 1:** Detailed inclusion and exclusion criteria.

Inclusion criteria	Exclusion criteria
Age ≥ 18	Previously received treatment for breast cancer
Newly diagnosed breast cancer, stage 0, I, II, or III	Patients receiving neoadjuvant therapy
After surgical resection for women with newly diagnosed breast cancer	Metastatic disease
Planned to receive endocrine therapy (± radiation therapy, ± chemotherapy), AND ≥ 7 days between approach and starting treatment	Adults unable to consent
Women recommended for endocrine therapy related to breast cancer	Male
Mini-mental status exam (MMSE) > 25 at baseline	MMSE ≤ 25 at baseline
English speaking	Pregnant or nursing women

**Table 2:** Observed ineligibility reasons.

Reason	N, (%)
Not a newly diagnosed breast cancer	63, (18.3)
Not recommended for endocrine therapy	56, (16.3)
Started neoadjuvant chemotherapy	46, (13.4)
Second opinion only	34, (9.9)
Started ET or without 7-day window for baseline	34, (9.9)
Stage 4 breast cancer (metastatic)	33, (9.6)
Other	32, (9.3)
Psychiatric diagnosis*	17, (4.9)
Stage 0 (DCIS)*	15, (4.4)
Patient denied surgical resection	3, (0.9)
Patient refused ET	3, (0.9)
Study incompatible with occupation	3, (0.9)
English not primary language	3, (0.9)
Unable to give consent	2, (0.6)
MMSE ≤ 25	0, (0)

Endocrine therapy (ET), ductal carcinoma in situ (DCIS). Data presented as number and % of ineligible.

*Eligibility criteria that were modified and subsequently allowed into the study.

### Measures

#### Perceived cognition

Assessment of perceived cognition was conducted via EMA self-report surveys. Multiple dimensions of perceived cognition were assessed: (1) frequency of perceived cognitive impairments (proportion of days in which participants reported discrete episodes of memory or attention impairment), (2) perceived impairment severity (continuous ratings of perceived forgetfulness or distraction), (3) impact of perceived impairment on quality of life (continuous ratings of the degree to which perceived impairments bothered or disrupted daily activities), and (4) perceived cognitive ability (continuous ratings of perceived mental clarity, concentration, speed, and focus). Episodes of perceived cognitive impairment were assessed once daily during the Bedtime survey, with participants asked to select which (if any) impairments they experienced that day from survey items containing exemplars of memory and attention impairments. Perceived impairment severity was assessed via 2 items presented 5 times daily, during all Beeped and Bedtime surveys. Example Perceived Cognition EMA survey items can be found in Supplementary Figure 1.

Participants were asked to indicate the degree to which they felt forgetful or easily distracted, using a 100pt visual analog scale (slider) from *not at all* to *extremely*. Impact of perceived impairment on quality of life was assessed via four items presented during the Bedtime surveys. Participants were asked to indicate the degree to which forgetting and distraction bothered them or disrupted their daily activities, again using a 100pt visual analog scale from *not at all* to *extremely*. Perceived cognitive ability was assessed via 4 items presented during each EMA survey (6 times daily). Participants were asked to indicate their perceived ability to focus, concentrate, think clearly, and think quickly, again using a 100pt visual analog scale from *not at all* to *extremely*. Selection of items and dimensions of perceived cognition was guided by existing instruments including the FACT-Cog^21^, cognitive failures questionnaire^22^, PROMIS Global Cognition^23^, and recent EMA studies of cognitive outcomes in breast cancer survivors^18,19^.

#### Psychosocial factors

Assessment of stress, pain, fatigue, worry, happiness, and sadness was conducted via the EMA self-report surveys. Each item was administered during Beeped and Bedtime Surveys, totaling 5 daily administrations. Participants were asked to rate the degree to which they were experiencing each item, using a 100 pt visual analog scale from *not at all* to *extremely*. Selection of each indicator was guided by previous EMA studies of affect variance^19^.

#### Performance-based measures of cognition

Participants were asked to complete 3 performance-based ambulatory cognitive assessments (“tasks”) at the end of Wake-up and Beeped Surveys, totaling 5 administrations per day (up to 35 administrations per burst). Each task was delivered in an ultra-brief format and took approximately 1 min to complete. We selected tasks assessing cognitive domains known to be sensitive to cancer and cancer treatment^24,25^, cognitive aging^26^, and risk for Alzheimer’s disease and related dementias^16,27^.

The Symbol Search task, measuring processing speed/attention, is a speeded two-alternative forced choice task where participants are asked to select a sample tile from the bottom of the display that matches one of the test tiles presented at the top of the screen^28,29^. The primary outcome of the Symbol Search task is median response time (RT) for accurate trials. The Colored Squares task, measuring visual working memory capacity involves a visual array change detection procedure where participants are asked to determine whether a single, colored square presented during the test phase is the same or different color than the square presented at the same location during the study array^30^. The primary outcome of the Colored Squares task is estimated visual working memory capacity (*k-score*, sensitivity scaled by study set-size). The Shopping List task, measuring associative long-term memory, is a delayed recognition task where participants are asked to determine whether shopping list item-price combinations presented during the retrieval phase match the combinations judged during the price judgment (*study*) phase of the task. The primary outcome for the Shopping List task is the proportion of correct responses during the retrieval phase. See Supplementary Figure 2 for examples of each task.

#### Behavioral factors

Sleep was assessed via (a) a wrist-worn activity monitor (Actiwatch Spectrum devices, Philips-Respironics; Murrysville PA) with an on-wrist sensor, and (b) self-ratings of sleep duration, restoration, and perceived insomnia (difficulty falling asleep and sleep interruptions) obtained during EMA Wake-up Surveys. Patients were instructed to wear the Actiwatches at all times including overnight and only remove when bathing or swimming. Sleep data collected via actigraphy were recorded, downloaded (using Philips Actiware software version 6.0.4, Philips Respironics, 2017) at 30-s epoch intervals, and scored by two independent scorers using a validated method detailed elsewhere. In short, scorers individually set sleep intervals (≥ 20 min in duration) and determined the daily cut-point and number of valid days. Scorers then adjudicated each recording for inter-rater reliability and confirmed the number of valid days, number of sleep intervals and differences in sleep intervals greater than 15 min in duration. A valid day of sleep actigraphy was defined as having at least 20 h of on-wrist time (with the exception of the first and last study day), no off-wrist periods greater than or equal to 60 min within 10 min of the start or end of a nighttime sleep interval, and no sign of constant false activity due to device battery failure^6,31^. Nighttime sleep parameters were calculated on the sleep interval with the longest duration overlapping the hours between 10PM and 8AM each day. The within-person mean and standard deviation were computed for all nighttime sleep variables across valid days.

#### Sleep parameters analyzed included the following variables

Number of Valid Days: The total number of valid sleep actigraphy days.

Sleep Midpoint: The midpoint of sleep was measured as the time half-way between the start (sleep onset) and end (sleep offset) of a nighttime sleep interval. The mean and variability, measured by standard deviation, of Sleep Midpoint (military time) was analyzed.

Total Sleep Time (TST): Total sleep time was measured by the number of hours of sleep in the nighttime sleep interval and does not include minutes of WASO. The mean and variability, measured by standard deviation, of TST were used.

Wake After Sleep Onset (WASO): WASO, a measure of sleep quality, was calculated as the number of minutes spent awake during the nighttime sleep interval.

Physical activity was measured utilizing hip-worn tri-axial movement monitoring devices (Actigraph GT3X, Actigraph, Pensacola, FL). Participants were asked to wear the Actigraph devices during waking hours only. Physical activity data collected at a rate of 80 Hz via actigraphy was converted to 3-axes and vector magnitude activity counts at a 60-s epoch length. Vector-magnitude cut points were based on Keadle’s Women’s Health parameters^32^. Nonwear periods were calculated in Actilife (version 6.13.4) based on Troiano 2007 Wear Time Validation Parameters, and then manually adjusted to confirm accuracy with data collection periods^33^. Data collected outside of the study data collection period (i.e. travel time or non-compliance wearing the device 8+ days) was verified with the study collection period windows and manually designated as “non-wear” within the “Wear Period Validation” window. A valid day of physical activity actigraphy was defined as wearing the Actigraph hip device for a minimum of 4 h per day. Time spent in three levels of physical activity (sedentary, light, moderate) were first summed for each day and then averaged over the 7-day measurement burst. Percentage of time spent in in each category of activity (sedentary/light/moderate) was calculated by dividing each respective activity category by the sum total of sedentary + light + moderate activity minutes for each data collection period).

#### Physical activity parameters analyzed included the following variables:

Valid Days: the total number of valid physical activity actigraphy days.

Wear Time: average number of hours Actigraph was worn per day divided by the number of Valid Days.

Steps Per Day: the average number of steps calculated by the sum of steps counted during scored time divided by the number of Valid Days.

Sedentary Time: percentage of total time in minutes of sedentary (0–199 counts per minute (CPM)) per total data collection period.

Light Activity Time: percentage of total time in minutes of light activity (200–2689 CPM) per total data collection period.

Moderate Activity Time: percentage of total time in minutes of moderate activity (≥ 2690 CPM) per total data collection period).

### Statistical analysis

Statistical analyses were conducted in SAS 9.04 program. To determine whether participants who withdrew differed from those who completed the study, two-sample t-tests and Wilcoxon-Ranked tests were conducted to compare demographic and outcome data collected during the baseline measurement burst. Indices of participation included: Screening rate, the proportion of new breast cancer patients meeting initial eligibility criteria of total patients screened in electronic medical records; consent rate, the proportion of patients that gave written informed consent of those approached during recruitment; study completion rate, the proportion of participants who did not withdraw (voluntarily or due to changes in study eligibility, such as change of therapy or loss to follow up) of the number who gave written informed consent; compliance rate, the number of measurement bursts completed by active participants at each wave.

An EMA survey was considered valid if participants completed all self-report survey items administered during that session (e.g., all affect and perceived cognition items administered during a Beeped survey). An arbitrary completion threshold of 50% of expected surveys was used as criteria for a ‘completed’ burst. Data collected via EMA were aggregated and scored as follows: Proportion of days with *perceived cognitive impairment*, the proportion of days in which a participant endorsed experiencing at least 1 focal impairment during the Bedtime Survey of total valid Bedtime Surveys for the Baseline measurement burst; *perceived impairment severity*, average of the two impairment severity items over all valid Beeped and Bedtime Surveys (higher values = more severe impairment); *impact of perceived impairments* on quality of life, average of the four impact items over all valid Bedtime Surveys (higher values = greater impact on quality of life); *perceived cognitive ability*, average of the four ability items over all valid Wake-up, Beeped, and Bedtime Surveys (higher values = greater perceived ability); mean rating for each psychosocial factor, average rating over all valid Beeped and Bedtime Surveys administered during the Baseline measurement burst. Ambulatory cognitive assessment data was considered valid if participants completed all three cognitive tasks administered within a given Survey (“session”). Performance-based cognitive assessments were first scored at the level of the individual session and then averaged over all valid administrations.

## Results

**Figure 2:**
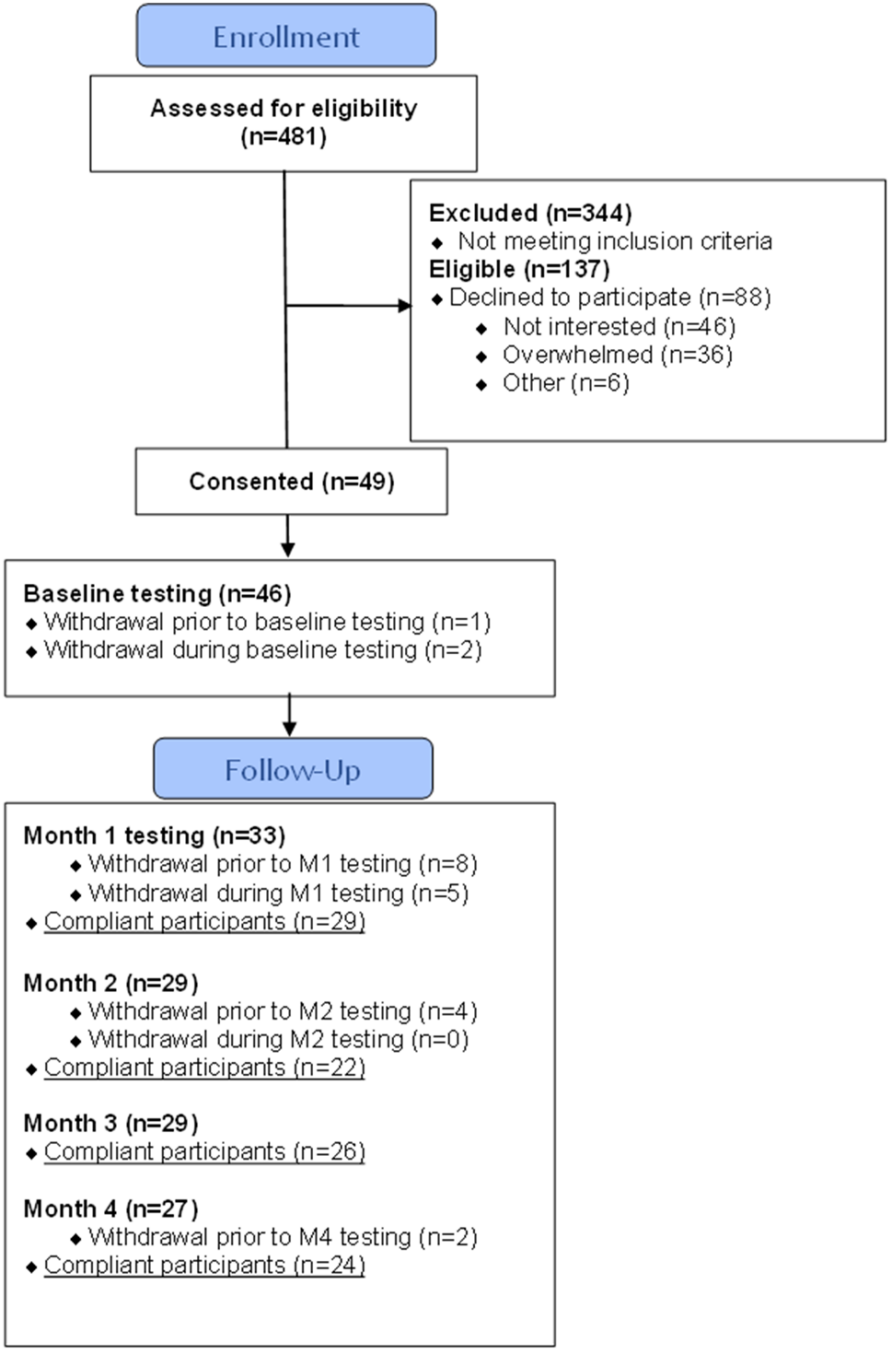
Consort Diagram for the study. A total of 137 potential participants were eligible out of a total 481 screened. Of the 137 approached 49 consented and 27 completed the study.

Over 18 months, 481 new breast cancer patients were screened (Fig. 2). Of those screened, 28% were eligible for the study (~ 8 women eligible per month of screening). The top three reasons for ineligibility were 1) not a new diagnosis of breast cancer, 2) not recommended for ET, or 3) recommended for neoadjuvant therapy (Table 2). Consent was obtained from 36% of eligible breast cancer patients. The study population was predominantly White, overweight, non-Hispanic, working, postmenopausal, and married. The predominant clinical characteristics included Stage I breast cancer, partial mastectomy surgical intervention, and radiation in addition to anastrozole ET (Table 3). The main reasons for declining participation were: 1) not interested in research, and 2) concerned about time and being overwhelmed. Of 49 women who consented to participate in the study, 19 voluntarily withdrew and 3 were withdrawn by the coordinator as lost to follow-up. Half of the withdrawals occurred after the baseline measurement burst (n = 11) and another 41% occurred after Month 1 (n = 9). Reasons for withdrawal were 1) too busy or overwhelmed (n = 11), 2) stopping ET by personal choice or physician recommendation (n = 4), 3) failure to return calls by study staff (n = 3), 4) concern for COVID-19 (n = 2) and 5) other (n = 2).

**Table 3:** Demographics and clinical characteristics.

Variable	All N = 49	Withdrawn N = 22	Completers N = 27	*P* value
Age (years)	60 ± 9.3	57 ± 7.9	62 ± 10.0	0.10
BMI (kg/m^2^)	29.6 ± 5.8	28.9 ± 7.1	30.2 ± 4.5	0.47
Race				
White	44 (90%)	20 (90.1%)	24 (89%)	0.91
Ethnicity				
Not Hispanic/Latino	44 (90%)	20 (90.1%)	24 (89%)	0.82
Working status (n = 47)				
Full-time	21 (45%)	8 (40%)	13 (48%)	0.81
Part-time	10 (21%)	5 (25%)	5 (19%)	
Homemaker	16 (34%)	7 (35%)	9 (33%)	
Type of work				
Professional	15 (31%)	6 (27%)	9 (33%)	0.55
Manager/Sales/Operative	11 (22%)	7 (32%)	4 (15%)	
Unemployed	6 (12%)	2 (9%)	4 (15%)	
Other	17 (35%)	7 (32%)	10 (37%)	
Household income (n = 46)				
< 50 K	16 (35%)	6 (30%)	10 (39%)	0.91
50–100 K	12 (26%)	5 (25%)	7 (27%)	
100–150 K	10 (22%)	5 (25%)	5 (19%)	
150 K+	8 (17%)	4 (25%)	4 (15%)	
Education (n = 47)				
High school—associate’s	19 (40%)	8 (40%)	11 (42%)	0.82
Bachelor’s	14 (30%)	7 (35%)	7 (26%)	
Graduate + (Master’s/MD/PhD)	14 (30%)	5 (25%)	9 (32%)	
Marital status				
Married/partner	36 (75%)	18 (86%)	18 (67%)	0.17
Single/divorced/widowed	12 (25%)	3 (14%)	9 (33%)	
Cancer stage				
Stage 0	7 (14%)	5 (22%)	2 (7%)	0.35
Stage I	32 (65%)	14 (64%)	18 (67%)	
Stage II	9 (19%)	3 (14%)	6 (22%)	
Stage III	1 (2%)	0	1 (4%)	
Cancer treatment				
ET	14 (29%)	9 (41%)	5 (18%)	0.13
RT + ET	28 (57%)	12 (54%)	16 (60%)	
CT ± RT + ET	4 (8%)	0	4 (15%)	
Refused ET	3 (6%)	1 (5%)	3 (6%)	
Menopausal status				
Pre-Menopausal	11 (23%)	6 (27%)	5 (18%)	0.73
Post-Menopausal	35 (71%)	15 (68%)	20 (74%)	
Peri-Menopausal	3 (6%)	1 (5%)	2 (8%)	
Type of endocrine therapy				
Anastrozole	29 (6%)	11 (52%)	18 (72%)	0.21
Letrozole	6 (13%)	2 (10%)	4 (16%)	
Tamoxifen	11 (24%)	8 (38%)	3 (12%)	

Categories with less than n = 49 are due to incomplete EMR data or missing questions on demographic forms.

Endocrine therapy (ET), Radiation therapy (RT), Chemotherapy (CT), Estrogen Receptor (ER), Progesterone Receptor (PR), Human Epidermal growth factor Receptor 2 (Her2). Data presented as mean ± SD and number, %. *P < 0.05.

Study compliance (the proportion of active study participants at each wave of data collection who completed the scheduled measurement burst) was 94% (46/49) at the baseline measurement burst, 88% at Month 1 follow-up (29/33), 76% at Month 2 (22/29), 90% at month 3 (26/29), and 89% at month 4 (24/27). Among participants who completed the study (N = 27), 89% of scheduled measurement bursts were completed (123 completed/139 total scheduled bursts). Average length of the study observation period for a participant was 215 days.

There were no significant differences in demographic or breast cancer clinical characteristics between women who completed the study and those who withdrew (Table 3). Group differences were observed in completeness of baseline data. Withdrawn participants provided fewer (p < 0.05) total valid days of data for EMA surveys, ambulatory cognitive assessments, sleep, and physical activity (Table 4). No other significant differences between groups were observed.

**Table 4:** Baseline mean differences between patients that withdrew and completed the study.

Variable (mean or %, ± SD)	All N = 49	Withdrawn N = 22		Completers N = 27	P-value
Smart phone data	*N* = *46*	*N* = *16*	*N* = *27*		
EMA Surveys Completed (%)	79.1 ± 23.3	66.3 ± 25.7	88.2 ± 16.7		< 0.01*
Ambulatory Assessments Completed (%)	78.3 ± 23.3	65.9 ± 26.4	87.0 ± 16.3		< 0.01*
Perceived cognition					
Cognitive Ability (AU)	76.3 ± 16.0	74.2 ± 16.7	77.7 ± 15.7		0.48
Days impairment reported (%)	0.21 ± 0.30	0.26 ± 0.37	0.16 ± 0.23		0.30
Impairment Severity (AU)	17.8 ± 16.9	20.3 ± 17.3	16.0 ± 16.6		0.40
Impairment Impact (AU)	14.7 ± 17.9	19.2 ± 22.9	11.5 ± 12.9		0.21
Objective cognition					
Symbol Search (*RT*)	1814 ± 567	2011 ± 792	1675 ± 271		0.09
Change Detection (*k-value*)	3.1 ± 0.6	3.0 ± 0.7	3.3 ± 0.5		0.17
Shopping List (*# accurate trials*)	7.8 ± 1.0	7.7 ± 1.0	7.9 ± 0.9		0.44
Affect (AU)					
Worried	19.6 ± 20.1	22.4 ± 21.9	17.7 ± 18.9		0.46
Happy	71.7 ± 17.4	73.9 ± 12.4	70.1 ± 20.3		0.44
Fatigue	36.1 ± 22.5	36.9 ± 22.1	35.6 ± 23.1		0.84
Stress	25.8 ± 23.4	27.2 ± 21.9	24.9 ± 24.9		0.75
Sad	18.8 ± 19.8	19.1 ± 17.6	18.6 ± 21.6		0.94
Pain	18.1 ± 19.8	18.7 ± 19.5	17.7 ± 20.3		0.86
Nighttime sleep	*N* = *45*	*N* = *19*	*N* = *26*		
Valid days	6.3 ± 2.2	5.1 ± 2.3	7.1 ± 1.8		< 0.01*
Sleep Midpoint (military time, h)	02:54 ± 01:00	02:54 ± 01:12	02:54 ± 00:54		0.93
Sleep Midpoint Variability (military time, h)	00:37 ± 00:17	00:39 ± 00:20	00:35 ± 00:166		0.84
Total Sleep Time (h)	7.6 ± 1.0	7.6 ± 1.3	7.7 ± 0.8		0.87
Total Sleep Time Variability (h)	1.1 ± 0.5	1.4 ± 0.6	1.0 ± 0.4		0.07
WASO (min)	44.2 ± 21.7	43.9 ± 19.5	44.5 ± 23.5		0.88
Physical activity	*N* = *45*	*N* = *18*	*N* = *27*		
Valid Days	6.5 ± 2.1	5.4 ± 2.2	7.2 ± 1.8		< 0.01*
Wear Time (h/day)	13.9 ± 2.7	13.6 ± 2.6	14.2 ± 2.7		0.35
Steps per day	6638.2 ± 4696.6	7139.6 ± 4500.1	6303.9 ± 4878.3		0.51
Sedentary Time (%)	70.6 ± 9.4	69.3 ± 8.5	71.6 ± 9.9		0.43
Light Activity Time (%)	28.2 ± 8.9	29.1 ± 8.4	27.5 ± 9.4		0.57
Moderate Activity Time (%)	1.2 ± 2.0	1.6 ± 3.0	0.92 ± 0.93		0.26

Standard Deviation (SD), Wake After Sleep Onset (WASO), Arbitrary Units (AU) on 100 point visual analog scale.

*P < 0.05.

## Discussion

Cancer and cancer treatments have been shown to accelerate cognitive decline^25,34–38^. While the prevalence of subjective cognitive decline in adults older than 45 years of age is ~ 11%^39^, up to 75% of cancer survivors report experiencing subjective cognitive decline symptoms^25,34,36,40,41^. The use of an ambulatory cognitive assessment approach can sharpen detection of subjective and objective cognitive decline symptoms, which are often momentary and periodic. Understanding the circumstances surrounding episodes of impairment is crucial to understanding the drivers and etiology of cancer- and cancer treatment-associated cognitive impairments. Therefore, we conducted a signal-finding study in women with breast cancer receiving ET to: (1) determine acceptability of such longitudinal mobile assessments, (2) assess characteristics of patients who completed the study versus those who did not, and (3) contextualize our experience to guide future studies that incorporate similar methods. Overall, we observed (1) study eligibility and consent rates of 28% and 36%, respectively, (2) no clinical or demographic differences between participants who completed the study and those who did not, but differences might have been difficult to detect given the sample size, (3) the majority of patients who did not complete the study withdrew early and were significantly less compliant to data collection at baseline, and (4) participants who did not complete the study did not appear to have differences in sleep or physical activity habits, nor were differences in cognition observed but, again, sample size makes it difficult to detect differences that are not large.

The rationale for the study design was to dissociate potential drivers of cognitive impairments experienced by newly diagnosed breast cancer patients over the course of their daily lives. Hypothesized drivers included ET effects themselves, factors found to correlate with patient reported cognitive outcomes in previous studies (psychosocial factors such as changes in stress, pain, and affect); behavioral factors known to influence cognition in studies of middle-aged and older adults (sleep and physical activity), and performance-based indicators of cognitive health (assessments of processing speed, working memory, and associative long-term memory). Our methodology involved the use of three different study devices simultaneously (smartphones, watches and hip actigraphy devices) for 7 days each month for several months. While this could have created a burden for a number of women, the most common reason for withdrawal was a global sense of stress and feeling overwhelmed. Indeed, stress from a cancer diagnosis is common for stage I-III cancer patients^42^. Stress levels are typically moderate to severe at the initial stages of diagnosis and remain high for approximately 6-months^42^. This suggests that the time-period chosen for this study was at the height of women being stressed over their disease.

No significant differences were observed in demographic or clinical characteristics between participants who completed the study versus those who did not complete. Participants who did complete the study were more compliant (i.e., used the devices the full 7 days, wore the devices longer, completed more cognitive surveys); these early data thus provide a predictor of who will complete the study. To our knowledge, this is the first study to assess the practicality of recruiting newly diagnosed breast cancer patients for a research study that utilizes a combination of technological devices (smartphones and multiple actigraphy devices). Breast cancer studies utilizing EMA approaches are limited. Existing studies have primarily focused on fatigue, physical activity and affect^18,43,44^.

Other elements of our study design and methodology are worth noting. As indicated in Fig. 1 and Table 1, we recruited women who would be receiving ET. However, at the time of study recruitment it was clinically ambiguous if a patient would move forward with chemotherapy, or radiation therapy. Given the flow of clinical care, it was impossible to restrict our enrollment to women who would be receiving ET as their only adjuvant therapy. Therefore, we designed the study to include a second baseline measurement after chemotherapy and before ET for women who received adjuvant chemotherapy. In practicality, only 8% of our study population received adjuvant chemotherapy, which is about half of the national average (19%)^45^.

In line with observations related to clinical care, 6% of women in our study refused ET even though it was initially recommended to them^46^. Future studies focused on recruiting women before starting ET may need to accommodate both the use of adjuvant therapy and ET refusal into sample size projections. Finally, our initial goal to recruit a homogenous group of cancer patients had to be revised to include women with a psychiatric diagnosis or DCIS, given their relatively high incidence in this population (Table 2)^47^.

In conclusion, it was difficult to identify demographic or clinical characteristics in women with breast cancer that could predict study completion, although initial compliance rate was indicative of study completion rate. Our experience suggests that retention might be improved by distributing data collection periods, developing more regular check-ins with patients, and using a “washout” period between study consent and baseline device use. Accounting for attrition between consent and enrollment or consent and study completion is an important aspect of sample size determination. Further, investigators should be aware of the clinical flow of treatment decisions in relationship to the research time line and account for these clinical decisions in their study design. Thus, our observations may inform future trials.

### Supplementary Information

Supplementary Information.

## Data Availability

The datasets during and/or analysed during the current study available from the corresponding author on reasonable request.
