# Supplementary material for: Design and methods of the mobile assessment of cognition, environment, and sleep (MACES) feasibility study in newly diagnosed breast cancer patients
Source: Sci Rep. 2024 Apr 9;14:8338. doi: 10.1038/s41598-024-58724-1 (PMC11004176; doi:10.1038/s41598-024-58724-1)
Supplement: Supplementary file 1 — Supplementary Information. [file 41598_2024_58724_MOESM1_ESM.pdf]

Design and Methods of the Mobile Assessment of Cognition, Environment, and Sleep (MACES) Feasibility Study in Newly Diagnosed Breast Cancer Patients

Rebecca Derbes, MPH[1], Jonathan Hakun PhD[2,3,4,5*], Daniel Elbich PhD[2,5], Lindsay Master, MAS[6], Sheri Berenbaum PhD[3], Xuemei Huang, MD, PhD[2,5], Orfeu M. Buxton PhD[4,6], Anne-Marie Chang[6] PhD, Cristina I. Truica MD[7], Kathleen M. Sturgeon PhD, MEd, MS[1]

Figure 1. Example Perceived Cognition EMA Items.

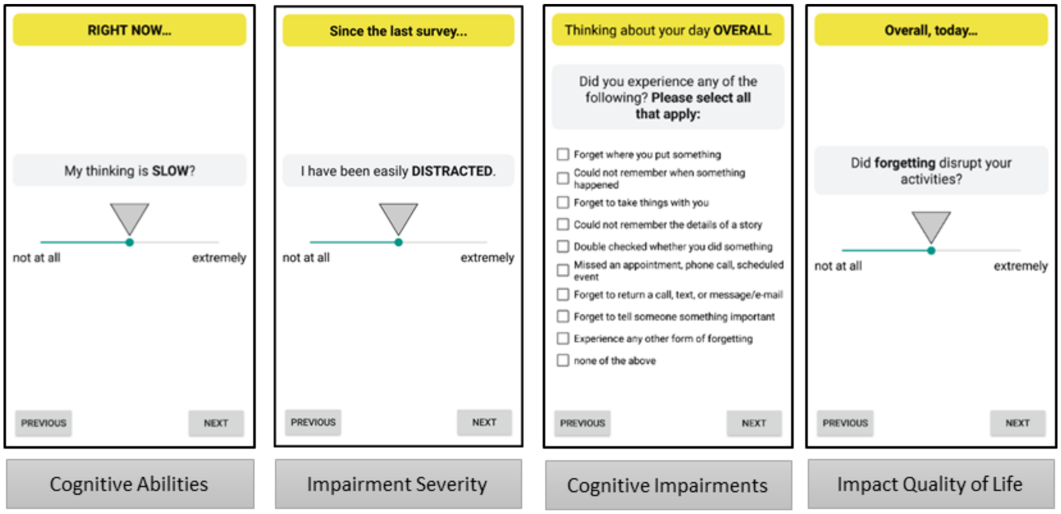

Momentary surveys of perceived cognitive ability and impairment severity were administered at every Beeped and Bedtime survey. Participants were asked to rate their perceived cognitive ability in the current moment and rate impairment severity since the previous survey. During Bedtime surveys participants were asked to catalog any specific impairments experienced during that day and rate the degree to which forgetting and distraction bothered or disrupted daily activities.

Figure 2. Ambulatory Cognitive Assessments.

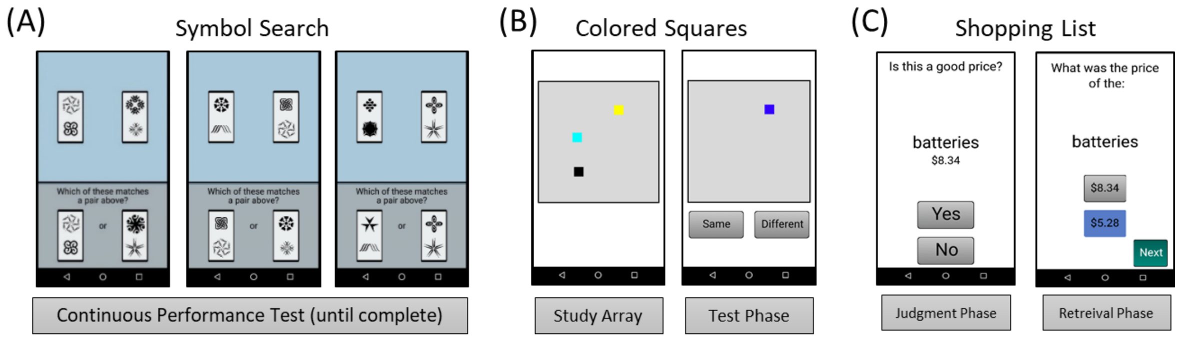

(A) Symbol Search task. (B) Colored Squares task. (C) Shopping List Task.
